# Long-horned Ceratopsidae from the Foremost Formation (Campanian) of southern Alberta

**DOI:** 10.7717/peerj.4265

**Published:** 2018-01-16

**Authors:** Caleb M. Brown

**Affiliations:** Royal Tyrrell Museum of Palaeontology, Drumheller, Alberta, Canada

**Keywords:** Ceratopsidae, Ornithischia, Dinosauria, Cretaceous, Campanian, Alberta, Horn, Cranial ornamentation, Display, Postorbital

## Abstract

The horned Ceratopsidae represent one of the last radiations of dinosaurs, and despite a decade of intense work greatly adding to our understanding of this diversification, their early evolution is still poorly known. Here, two postorbital horncores from the upper Foremost Formation (Campanian) of Alberta are described, and at ∼78.5 Ma represent some of the geologically oldest ceratopsid material. The larger of these specimens is incorporated into a fused supraorbital complex, and preserves a massive, straight, postorbital horncore that is vertical in lateral view, but canted dorsolaterally in rostral view. Medially, the supracranial sinus is composed of a small, restricted caudal chamber, and a large rostral chamber that forms the cornual diverticulum. This morphology is distinct from that of the long-horned Chasmosaurinae, and similar to, but still different from, those of younger Centrosaurinae taxa. The smaller specimen represents an ontogenetically younger individual, and although showing consistent morphology to the larger specimen, is less taxonomically useful. Although not certain, these postorbital horns may be referable to a long-horned basal (i.e., early-branching, non-pachyrhinosaurini, non-centrosaurini) centrosaurine, potentially the contemporaneous *Xenoceratops*, largely known from the parietosquamosal frill. These specimens indicate the morphology of the supracranial sinus in early, long-horned members of the Ceratopsidae, and add to our understanding of the evolution of the cranial display structures in this iconic dinosaur clade.

## Introduction

Ceratopsidae (horned dinosaurs) represents a diverse, abundant, and well-documented group of Late Cretaceous dinosaurs. This clade, characterized by a rostral bone, medial nasal horn/boss, pair of postorbital horns/bosses, and a parietosquamosal frill bearing peripheral epiossifications, is one of the last major radiations of non-avian dinosaurs prior to the end-Cretaceous mass extinction (Ryan, Chinnery-Allgeier & Eberth, 2010; Sampson & Loewen, 2010). Despite their late appearance, their iconic morphology and extensive fossil record spanning late Campanian through to late Maastrichtian has ensured their status as one of the most recognizable groups of dinosaurs. In contrast, their earlier record is much more limited (Kirkland & DeBlieux, 2010; Sampson & Loewen, 2010; Ryan, Evans & Shepherd, 2012; Lund et al., 2016). The lack of understanding regarding the early evolution of the Ceratopsidae arises from the rarity of known taxa from early in the evolution of the clade. This is highlighted by the Turonian age of *Zuniceratops christopheri*, the putative sister taxon (Wolfe et al., 2010), incurring a significant ghost lineage regarding the early diversification of this clade.

Within Ceratopsidae, two robustly supported sister clades have emerged, the Centrosaurinae which go extinct in the early Maastrichtian, and the Chasmosaurinae which persist to the end-Cretaceous mass extinction (Dodson, Forster & Sampson, 2004; Sampson & Loewen, 2010). Although diagnosed on a suit of cranial synapomorphies, the most conspicuous differences between these groups pertain to their cranial ornamentation. Previously, Centrosaurinae was typically characterized by a short squamosal, large nasal horn or boss, and large parietal spikes and hooks. In contrast, the Chasmosaurinae was typically characterized by a long squamosal, generally large postorbital horns, and more modest frill ornamentation.

The last decade has seen a dramatic increase in newly described species within both these groups (Ryan, Chinnery-Allgeier & Eberth, 2010; Sampson & Loewen, 2010). Of particular interest has been the discovery of multiple new species recovered as basal (i.e., early-branching, non-pachyrhinosaurini, non-centrosaurini) members of the Centrosaurinae, including *Albertaceratops nesmoi* (Ryan, 2007), *Diabloceratops eatoni* (Kirkland & DeBlieux, 2010), *Machairoceratops cronusi* (Lund et al., 2016), and *Nasutoceratops titusi* (Sampson et al., 2013). These taxa, as well *Zuniceratops christopheri* the putative sister taxon of Ceratopsidae (Wolfe & Kirkland, 1998; Wolfe et al., 2010), have illustrated that large postorbital horns likely represent the plesiomorphic condition for both Ceratopsidae, and Centrosaurinae specifically. In addition to these recent discoveries, other newly described taxa, such as *Xenoceratops foremostensis* (Ryan, Evans & Shepherd, 2012), and *Wendiceratops pinhornensis* (Evans & Ryan, 2015), are known from diagnostic parietals, but do not preserve postorbital horns. Our understanding of the early evolution of Chasmosaurinae, is more limited than Centrosaurinae, with few taxa known from prior to the late Campanian (Longrich, 2013).

Contrasting the diverse dinosaur assemblages documented from the other (later) units of the Late Cretaceous Belly River Group (i.e., Oldman and especially Dinosaur Park formations) of Alberta, the dinosaur assemblage from the Foremost Formation is more poorly represented. Despite microvertebrate assemblages revealing a reasonably diverse dinosaur fauna from the Foremost Formation (Baszio, 1997; Brinkman et al., 2004; Frampton, 2007; Cullen et al., 2016), named taxa, other than microvertebrate material, are limited to the pachycephalosaur *Colepiocephale lambei* and the centrosaurine ceratopsid *Xenoceratops foremostensis* (Schott, Evans & Williamson, 2009; Ryan, Evans & Shepherd, 2012). The hypodigm of *Xenoceratops* is largely restricted to elements of the parietosquamosal frill, and although a fragmentary skull (TMP 2010.076.0024) is potentially referable (see Ryan, Evans & Shepherd, 2012), much of the cranial and postcranial anatomy is unknown. Here, two isolated ceratopsid postorbital horncores are described from the Campanian Foremost Formation of Alberta, illustrating early evolution of massive postorbital horns within the ceratopsid radiation.

## Materials & Methods

Two isolated postorbital horncores are described. TMP 1989.068.0001 is a large, nearly complete left supraorbital complex, bearing the basal half of the large horncore. TMP 2017.022.0037 is a much smaller, isolated left postorbital horncore. TMP 2017.022.0037 was collected under an excavation and collection permit to CMB (17–017) provided by Alberta Culture and Tourism and the Royal Tyrrell Museum of Palaeontology. Both specimens are housed at the Royal Tyrrell Museum of Palaeontology, Drumheller, Alberta.

Photographs were taken using a Canon EOS 6D digital SLR camera with a 24–105 mm lens, with alternations (i.e., brightness/contrast adjustment and removal of background) performed in Adobe Photoshop CS5. Figures were prepared in Adobe Illustrator CS5. Measurements we taken to the nearest mm using digital calipers for measurements less than 150 mm, and a flexible tape measure for those over 150 mm and for circumferences.

## Results

### Systematic palaeontology

**Table:** 

**Ornithischia** Seeley, 1887, *sensu* Sereno, 1998
**Ceratopsia** Marsh, 1890, *sensu* Dodson, 1997
**Ceratopsidae** Marsh, 1888, *sensu* Sereno, 1998
Ceratopsidae indeterminate

### Locality and horizon

**Figure 1:**
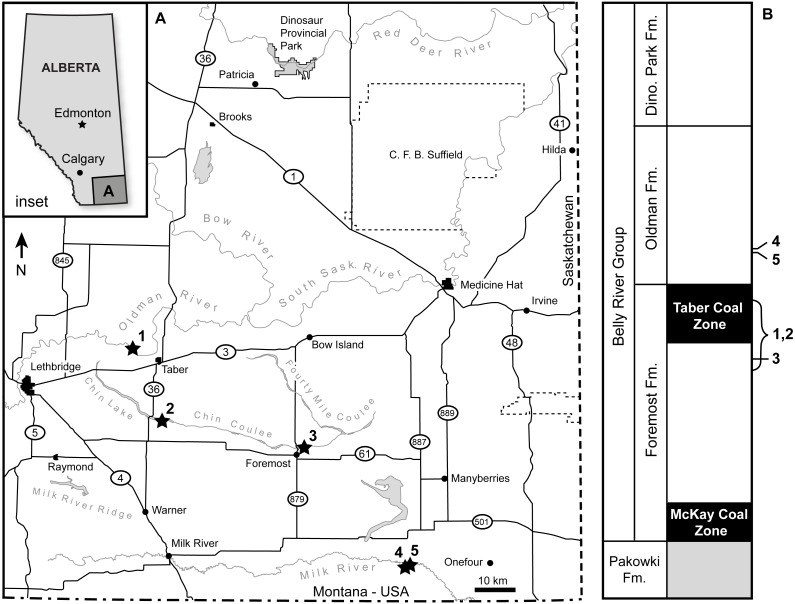
Geographic and stratigraphic occurrence of the specimens. (A) Geography of southern Alberta, showing the locations of both specimens described here, and relevant comparative specimens. (B) Geology for southern Alberta Belly River Group, showing occurrence of same specimens in A. (1) TMP 1989.068.0001, (2) TMP 2017.022.0037, (3) *Xenoceratops* type locality (Ryan, Evans & Shepherd, 2012), (4) *Wendiceratops* type locality (Evans & Ryan, 2015), (5) *Albertaceratops* type locality (Ryan, 2007).

**Figure 2:**
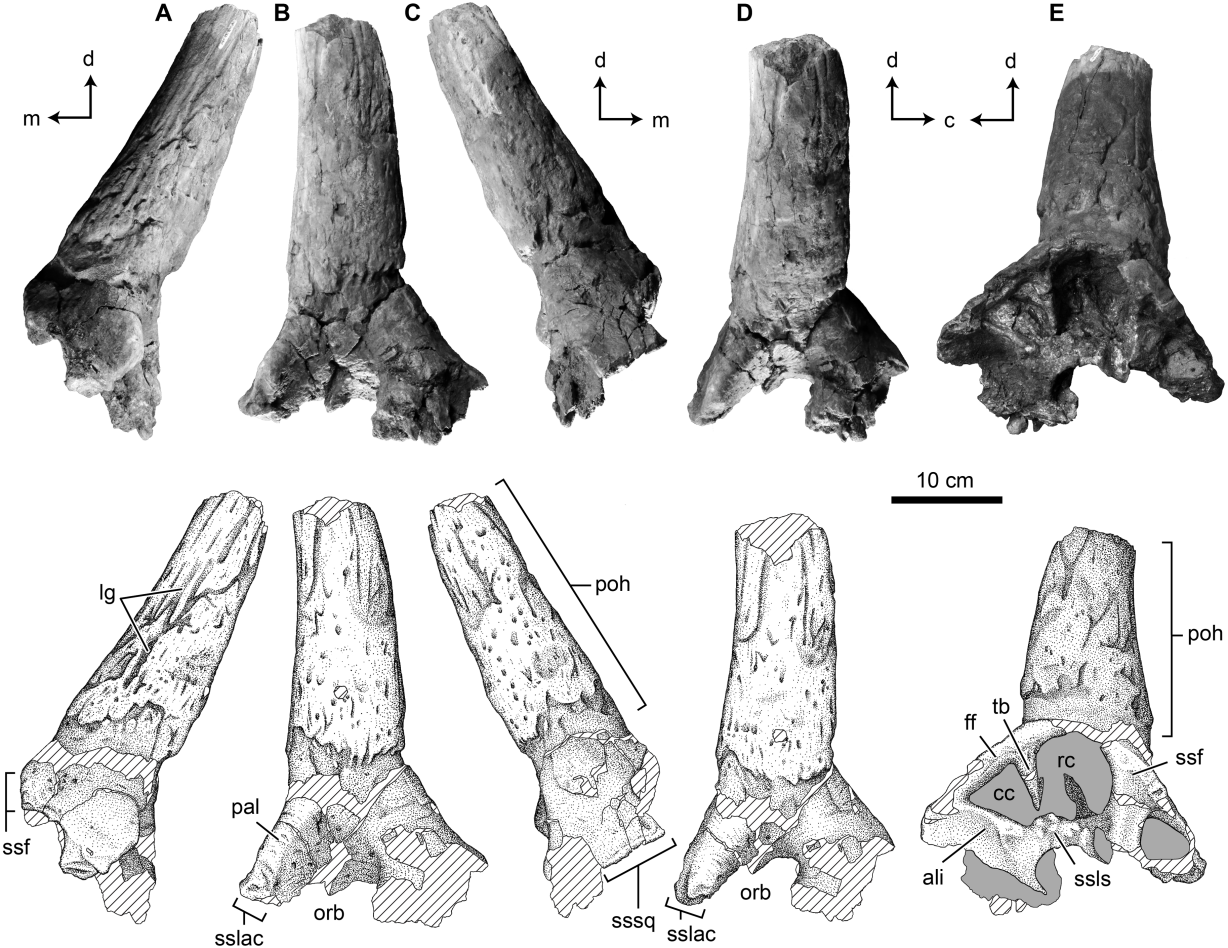
Photographs and interpretive scientific illustrations of the supraorbital complex TMP 1989.068.0001. TMP 1989.068.0001 in (A) rostral, (B) ventrolateral, (C) caudal, (D) lateral and (E) medial views. Hashed areas indicated broken bone surface, grey areas indicate rock matrix. Arrows indicate orthogonal anatomical orientations. For abbreviations see Anatomical Abbreviations and Orientation Abbreviations. Scale equals 10 cm.

TMP 1989.068.0001 was collected in 1989 from *in situ* Foremost Formation outcrop along the north side of the Oldman River, 8 km northwest of the town of Taber, Alberta (Fig. 1A-1). TMP 2017.022.0037 was surface collected along the south beach of Chin Lake during low water levels in the fall of 2017, 5 km east of the Highway 36 bridge (Fig. 1A-2). Outcrops at both locations comprise the uppermost Foremost Formation, dominated by the Taber Coal Zone (Russell & Landes, 1940; McLean, 1971) (Fig. 1B). Palynomorphs recovered with TMP 1989.068.0001 were stratigraphically non-informative (D Braman, pers. comm., 2016). Lithostratigraphically correlative ashes from sections at Kennedy Coulee MT have been radiometrically dated to ∼78.5 Ma (Goodwin & Deino, 1989). The specimens are penecontemporaneous with *Xenoceratops* from the upper Foremost Formation with a suggested age of 78 Ma (Ryan, Evans & Shepherd, 2012), and older than those of *Albertaceratops* and *Wendiceratops* from the lower Oldman Formation, which have been suggested to date between 78.7 and 79.0 Ma (Evans & Ryan, 2015). Current work reanalyzing radiometric dates for the Belly River Group will clarify the ages of these specimens, as well as those of other taxa. Detailed locality and conservation data for both specimens are on file at the Royal Tyrrell Museum of Palaeontology, Drumheller, Alberta.

### Description

#### TMP 1989.068.0001

The specimen represents indistinguishably fused left postorbital, palpebral, frontal and prefrontal bones from a ceratopsid dinosaur (Fig. 2). Together the postorbital and palpebral preserve the dorsal half of the orbit, with a rostrocaudal diameter of ∼101 mm (Figs. 2B and 2D, Table 1).

**Table 1:** Linear measurements of the postorbital horncores TMP 1989.068.0001 and 2017.022.0037 and comparative ceratopsid and non-ceratopsid taxa.

**Taxon**	**Specimen**	**Postorbital horncore**				**Orbit**
		**Basal circ.**	**Rostrocaudal diameter**	**Transverse diameter**	**Rectalinear height**	**Rostrocaudal diam.**
Indeterminate	TMP 1989.068.0001	384	117	107	306^a^	101
Indeterminate	TMP 2017.022.0037	280	75	64	148^a^	–
*Zuniceratops*	MSM P2101	150	51	33	88	–
*Zuniceratops*	MSM P2103	190	73	42	207	–
*Turanoceratops*	ZIN PH 1968/16	–	60	47	140	–
*Albertaceratops*	TMP 2001.026.0001	350/357	137/139	77/81	431/-	100/-
*Diabloceratops*	UMNH VP 16699	328/-	106/	102/-	255/-	-/-
*Nasutoceratops*	UMNH VP 19466	364/382	116/142	107/111	457	-/-
*Centrosaurus*	Sample mean (sd, *n*)	232 (51,24)	83 (16,26)	66 (13,25)	77 (34,27)	95 (13,26)
*Chasmosaurus*	Sample mean (sd, *n*)	268 (76,32)	94 (30,32)	75 (21,32)	131 (88,32)	97 (13,16)
*Agujaceratops*	Sample mean (sd, *n*)	231 (103,13)	87 (27,13)	70 (19,13)	248 (72,13)	–
*Arrhinoceratops*	Sample mean (sd, *n*)	390 (68,2)	–	–	428 (25,2)	103 (26,2)
*Anchiceratops*	Sample mean (sd, *n*)	450 (22,5)	132 (8,2)	98(26,20)	484 (116,5)	103 (14,4)

**Notes.**

a Denotes incomplete measure due to breakage. All measurements in mm.

##### Palpebral.

The palpebral is preserved in its entirety, and shows distinct, open sutural contacts with the lacrimal rostroventrally and a fused and indistinguishable contact with the postorbital caudally, and prefrontal medially. The palpebral forms the arcuate rostrodorsal margin of the orbit and shows a relatively conserved ceratopsid morphology (Fig. 2B). It is of consistent dorsoventral thickness along most of its rostrocaudal length (∼25 mm), but shows a slight thickening caudally where it contacts the postorbital (∼35 mm). Transversely the element also thickens dorsoventrally towards its medial extreme. The dorsal and ventral (forming the rostrodorsal wall of the orbit) surfaces of the palpebral are smooth, whereas the lateral surface of the rounded orbital rim bears slight rugosities. The rostral buttress is rounded with no distinct ornamentation.

##### Postorbital.

The postorbital bone is largely complete and forms the dorsal and caudal margins of the orbit, where the surface is smooth and perforated by occasional foramina (Figs. 2B and 2D). It is indistinguishably fused to the palpebral rostrally, frontal medially, and prefrontal rostromedially, thought this last element is unclear due to poor preservation. The caudoventral margin of the element is both incomplete and distorted, and the contact with the jugal is not preserved. Caudally a small portion of the suture for the squamosal is preserved, but much of the contact is lost (Fig. 2C). The medial surface of the dorsal margin of the orbit preserves an open and complex sutural contact for the laterosphenoid (Fig. 2E). Medially, the caudal portion of the postorbital bears a shallow alisphenoid fossa, whereas the rostral portion shows deep and prominent fossae associated with the supracranial sinus.

##### Supracranial (cornual/frontal) Sinus.

The supracranial sinus (sensu Farke, 2010) (i.e., frontal sinus, sensu Forster, 1996; Farke, 2006) is well developed, and although still partially infilled with matrix, it penetrates deep laterally within the postorbital, and dorsolaterally into the postorbital horncore (Fig. 2E). Further medially, the sinus is roofed by the frontal, but the exact composition cannot be determined, as the sutures are not visible. In the postorbital region, and lateral portion of the frontal region of the supracranial sinus there is a vertically oriented transverse buttress (or strut) dividing this area into rostral and caudal chambers (sensu Farke, 2010). The transverse buttress projects ventrally from the roof of the sinus, but does not span the entire dorsoventral height of the sinus, rather it terminates 8 mm from the sinus floor, incompletely separating the two chambers. The transverse buttress is wedge-shaped, being 21 mm thick dorsally where it contacts the sinus roof, and taping to a point 47 mm further ventrally.

The transverse buttress is not centered ventral to the horncore as in all other ceratopsids (Farke, 2010), but is located caudal to the base of the horncore (Fig. 2E). As a result, the division is unequal, with the round rostral chamber of the supracranial sinus being twice the size of the triangular caudal chamber. At the level of the transverse buttress, the rostral chamber is 66 mm in rostrocaudal length and 75 mm in dorsoventral height. In contrast, the caudal chamber is 48 mm in rostrocaudal length and 53 mm in dorsoventral height.

The caudal chamber of the supracranial sinus is restricted to the main body of the caudal postorbital, whereas only the rostral chamber enters the postorbital contributing to the cornual diverticulum (sensu Farke, 2010) (i.e., cornual sinus, sensu Forster, 1996). The depth to which either chamber penetrates laterally, and how far the cornual diverticulum penetrates the horncore, is unknown, as both chambers remain infilled with extremely hard matrix. The minimum lateral penetration of the chambers (from the margin of the frontal fontanelle) is 86 mm for the rostral chamber and 43 mm for the caudal chamber. The partially prepared lateral extreme of the rostral chamber also shows a laterally offset secondary (accessory) transverse buttress which only partially bisects this chamber.

##### Postorbital Horncore.

The most obvious feature of the postorbital is the large postorbital horncore located dorsal to the orbit. The base of the horncore is positioned directly dorsal to the orbit, with its basal midpoint positioned only slightly caudal from the midpoint of the orbit. The rostral margin of the horncore base is positioned approximately in line with the postorbital-palpebral suture, while the caudal margin of the horncore base is positioned just caudal to the caudal wall of the orbit.

The horncore projects predominantly dorsolaterally, with no distinct rostral or caudal component, such that in rostral view (Fig. 2A) the horncore oriented at ∼45°, and in lateral view (Fig. 2D) the horncore is approximately vertical. The angle between the orbital rim, and the main axis of the horncore is 140°. This indicates that if the orbital rim is vertically positioned (as is generally the case in Ceratopsidae) the horncore projects dorsolaterally at an angle of approximately 45° to the horizontal plane.

The horncore is massive, having a basal circumference of 384 mm, with an incompletely preserved length of 306 mm (Table 1). This preserved length likely represents only the basal one-half of the total horncore length, with the distal half lost due to erosion. In cross-section, the horncore is nearly circular, with rostrocaudal (parasagittal) and transverse basal diameters of 117 and 107 mm, respectively, being nearly equal—and both exceeding the rostro-caudal diameter of the orbit. At the broken apex (306 mm above the base), these diameters are 88 and 74 mm, respectively, indicating both a slightly rostrocaudal ellipsoid shape, and a reduction of only one-quarter of the diameter at the point of break. In addition to being nearly circular, the horncore is straight (or negligibly curved), with no distinct curvature (either rostrocaudally or transversely) or torsion (Fig. 2). The base of the horncore is not flared, as seen in many other ceratopsids, but shows a relatively constant thickness. Similarly, the apical tapering of the horncore is gradual, with a steady decrease in circumference towards the apex.

The surface of the horncore is rugose, and dominated by distinct longitudinal grooves (Fig. 2). These grooves run sub-parallel along the element, and show occasional; branching apically. When observed, the basal terminus of the grooves is demarcated by a foramen, while apically the grooves become less distinct and gradually disappear. These grooves are up to 6 mm deep and occasionally greater than 100 mm long, traversing much of the preserved length of the horncore. Interspersed amongst the grooves are foramina that are not associated with superficial furrows. The bone texture between the groove is porous and rugose. The base of the horncore shows a distinct constriction, most prominent on the caudolateral and caudal aspects, where the magnitude is ∼10 mm. The rugose texture and longitudinal furrows dominating the majority of the horncore are noticeably absent basal to the constriction.

##### Frontal/Prefrontal.

Due to the fused nature of the sutures it is not fully clear, but the frontal appears to be preserved medial to the postorbital horncore and forms the dorsal roof over the medial portion of the supracranial sinus (Fig. 2E). Laterally its contact with the postorbital is indistinguishable, as is its more cranial contact with the prefrontal. Although only partially preserved, the medial aspect of the element truncates in a smooth and rounded horizontal shelf lying approximately in the parasagittal plane. This rounded edge demarcates the limit of the ∼20 mm thick frontal roof over the supracranial sinus, and indicates a large open frontal fontanelle (sensu Farke, 2010). Rostrally this rounded margin thickens (up to 38 mm), and develops vertically oriented ridges and grooves that likely represents a midline frontal-frontal suture. Ventrally the element forms the smooth dorsal roof of the supracranial sinus, with a well-developed transverse buttress. The frontal roof is not flat, but shows a distinct doming in the sagittal plane. It is unclear, but a portion of the rostral extent of the roof of the supracranial sinus represents the prefrontal. Little can be said of the morphology of the prefrontal, other than a small fossa, potentially a portion of the sinus, is preserved along its broken medial surface. This sinus is 43 mm in rostrocaudal length and 31 mm in dorsoventral height at its medial-most extreme, and remains infilled with matrix.

Alternatively to the interpretation above, it is possible that only the postorbital is preserved dorsomedially, and the rounded horizontal shelf exposed medially represents a portion of the postorbital-frontal suture. This is unlikely as this suture would occur further medially, and be much thinner and without the strong interdigitations seen in other ceratopsid taxa, and would also be distinct from the condition in TMP 2017.022.0037.

#### TMP 2017.022.0037

**Figure 3:**
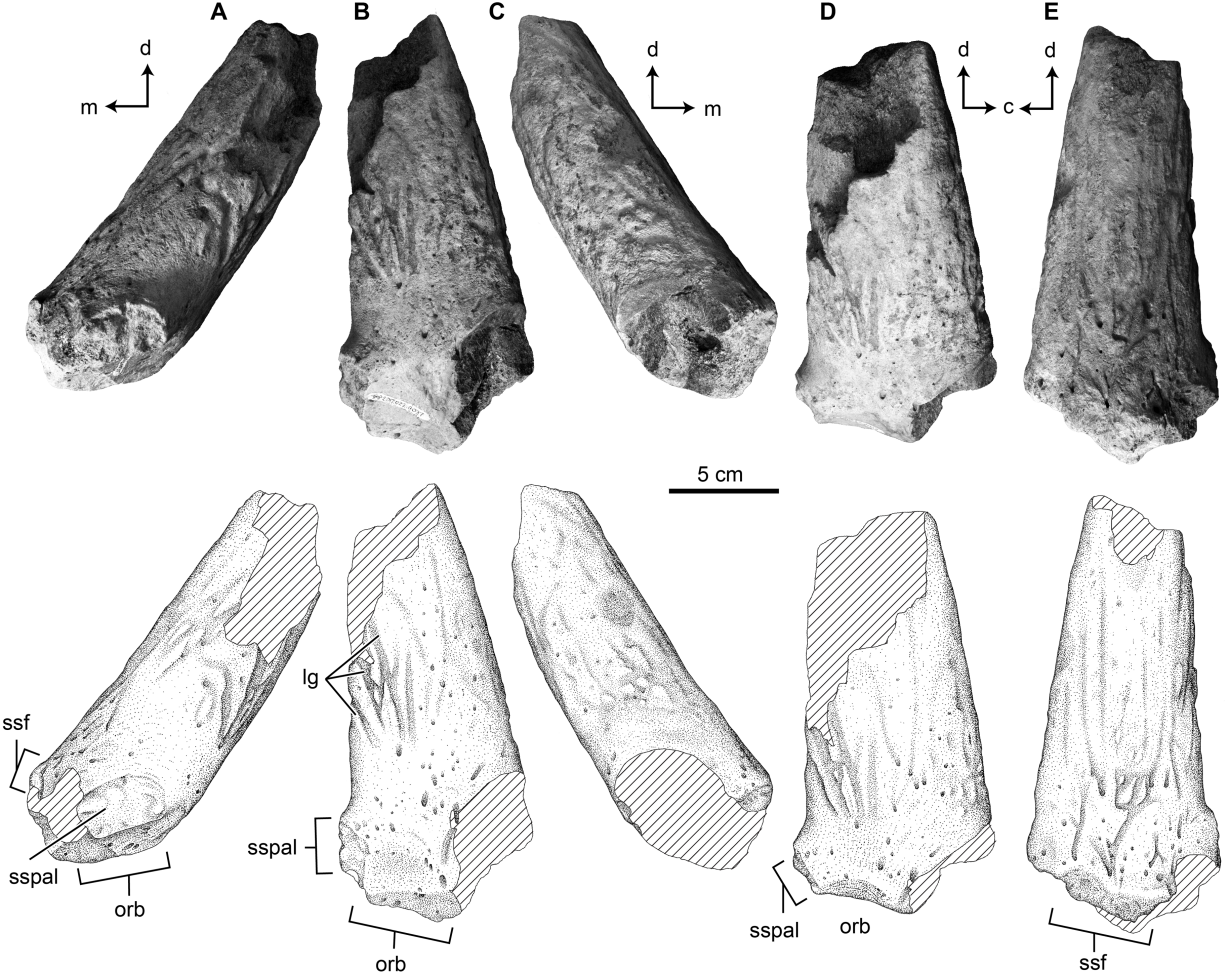
Photographs and interpretive scientific illustrations of the postorbital horncore TMP 2017.022.0037. TMP 2017.022.0037 in (A) rostral, (B) ventrolateral, (C) caudal, (D) lateral and (E) dorsomedial views. Hashed areas indicated broken bone surface. Arrows indicate orthogonal anatomical orientations. For abbreviations see Anatomical Abbreviations and Orientation Abbreviations. Scale equals 5 cm.

This specimen is both much smaller and less complete than TMP 1989.068.0001, and consists of the horncore base, open palpebral suture rostrally, a portion of the open frontal suture medially, and dorsal roof of the orbit (Fig. 3). The palpebral suture is reniform in cross section with a gently convex dorsal margin, concave ventral margin, and rounded lateral margin—forming the rostral margin of the orbit (Fig. 3A). Only a small portion of the frontal suture in preserved. The suture is located at the medial based of the horncore, and shows subtle vertically oriented ridges and grooves (Fig. 3E).

Due to incompleteness, the position of the horn is not entirely clear, but it appears to be centrally positioned over the orbit. As with TMP 1989.068.0001, the horncore projects dorsolaterally, but remains in the transverse plane (Figs. 3B and 3D). The orbital rim is not preserved enough to provide a vertical plane orientation, but in comparison to TMP 1989.068.0001, the angle between the orbital roof and the horncore is similar (145° and 159°), suggesting a similar angle of ∼45° to the horizontal (Figs. 3A and 3C).

The basal circumference is 230 mm, with a preserved length of 148 mm as the apex is lost (Table 1). In cross section, the horn is slightly more elliptical rostrocaudally than TMP 1989.068.0001, with rostrocaudal and transverse basal diameters of 75 and 64 mm, respectively. At the broken apex, these diameters are ∼47 and ∼54 mm, suggesting a slight change in cross-sectional shape along length, with a significant portion of the apical length missing. Although not complete, the horncore base is straight, with just a hint of a dorsomedial curvature towards the apex (Fig. 3C).

As with TMP 1989.068.0001, the surface of the horncore is dominated by distally branching, longitudinal grooves, foramina, and a porous and rugose texture. The distinct basal constriction on TMP 1989.068.0001 is not seen in this horncore, but the caudolateral base is missing.

A break across the caudal margin of the base exposes a hollow core, but this appears to represent a cancellous void space, rather than that finished surface of a cornual diverticulum of the supracranial sinus (i.e., cornual sinus).

### Comparisons

#### TMP 1989.068.0001

Despite sharing several similar features, the Foremost Fm. horncore TMP 1989.068.0001 is distinct from those of other described long-horned centrosaurine taxa. Other basal centrosaurines including *Albertaceratops* (TMP 2001.026.0001) and *Diabloceratops* (UMNH VP 16699*)*, as well as *Zuniceratops* (MSM P 21021), have a rostral component to their basal projection, whereas that of TMP 1989.068.0001 is oriented nearly vertical, with no distinct rostral or caudal component. Although positioned vertically in lateral view, the horncore is canted ∼45° dorsolaterally. This dorsolateral orientation is distinct from those of most centrosaurine taxa, in which the horncore is largely dorsally directed, with exceptions including *Nasutoceratops* (UMNH VP 16800), and *Coronosaurus* (TMP 2002.068.0005/0012) and MOR 692. Although both postorbital horncores are preserved in the holotype of *Albertaceratops* (TMP 2001.026.0001), the degree of lateral canting is difficult to assess due to transverse compression of the skull.

With approximately only the basal half of TMP 1989.068.0001 preserved, it is difficult to assess the overall curvature, but the preserved portion suggest a largely straight horncore. This is different from the condition seen in *Nasutoceratops* (UMNH VP 16800), with broad rostrolateral and dorsal curvature, as well as *Albertaceratops* (TMP 2001.026.0001) and *Diabloceratops* (UMNH VP 16699), which show rostral and caudal curvatures, respectively. The rostral curvature in *Albertaceratops* is largely restricted to the apical half, however, so a similar distal curvature in TMP 1989.068.0001 cannot be ruled out.

The basal cross-section of TMP 1989.068.0001 is nearly circular, distinct from the subtriangular cross section in *Diabloceratops* (UMNH VP 16699), and the rostrocaudally elongate cross section seen in *Albertaceratops* (TMP 2001.026.0001) and *Machairoceratops* (UMNH VP 16800). The distinct constriction at the base of the horn is similar to that seen in *Albertaceratops* (TMP 2001.026.0001). Both the position of this constriction, and the loss of the rugose texture and grooves suggest that this marks the basal extent of the horn sheath, as seen in some extant bovids (Ryan, 2007).

**Figure 4:**
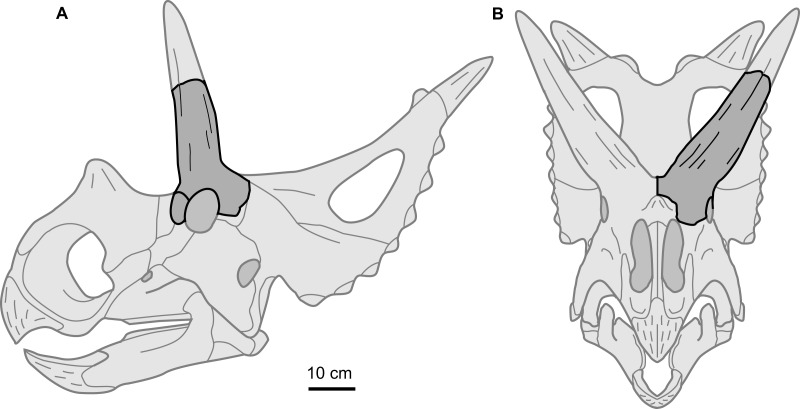
Supraorbital complex TMP 1989.068.0001 projected onto the skull of a representative basal centrosaurine. Reconstruction of generalized centrosaurine skull (light grey) in (A) left lateral, and (B) rostral views, showing relative position of the isolated supraorbital complex TMP 1989.068.0001 (dark grey). Skull reconstruction based on parietal of *Xenoceratops* (Ryan, Evans & Shepherd, 2012), and the face of *Wendiceratops* (Evans & Ryan, 2015) and *Diabloceratops* (Kirkland & DeBlieux, 2010). Scale bar = 10 cm.

Perhaps the most notable feature of the supraorbital is the large size of the postorbital horncore. In both basal dimensions and projected length, this horncore exceed those of most basal centrosaurines. The dimensions of the horncore are similar to *Nasutoceratops* (UMNH VP 19466), slightly exceed those of *Albertaceratops* (TMP 2001.026.0001), and greatly exceed those of *Diabloceratops* (UMNH VP 16699) (Table 1). For chasmosaurines, the overall size and proportions of the postorbital horncore are most similar to those of the *Arrhinoceratops brachyops* (ROM 797, 1439), and the larger *Anchiceratops ornatus* (ROM 802, CMN 8535, TMP 1983.001.0001, 1984.12.16). Despite the large size of the postorbital horncore, the rostrocaudal diameter of the orbit (101 mm) is within the range with those of the later, well-represented taxa *Centrosaurus* (mean = 95 mm, sd = 13.5, *n* = 26) and *Chasmosaurus* (mean = 96 mm, sd = 13.6, *n* = 14). This may suggest that the postorbital horncore does not derive from a larger animal, but rather is from a comparably sized animal, but with a relatively larger postorbital horncore (Fig. 4). This is consistent with the morphology seen in *Albertaceratops*, with a rostrocaudal orbit diameter of 100 mm (Table 1).

The presence of a fully roofed supracranial sinus in TMP 1989.068.0001 is distinct from the condition in the non-ceratopsid ceratopsoids *Zuniceratops* (MSM P3812) and *Turanoceratops* (ZIN PH 1968/16), which possess a frontoparietal depression that is not secondary roofed over by the frontal, lacks a transverse buttress, and does not penetrate the postorbital horn (Farke et al., 2009; Sues & Averianov, 2009a; Farke, 2010). The assignment to Ceratopsidae is based largely in the presence of this secondarily roofed supracranial sinus, which may represent a ceratopsid synapomorphy. If this proves not to be the case, TMP 1989.068.0001 may only be assignable to Ceratopsoidea. The incorporation of the supracranial sinus into the base of the postorbital horncore (forming the cornual diverticulum) is similar to the condition seen in many long-horned ceratopsids (Farke, 2006; Farke, 2010), including *Anchiceratops* (TMP 1984.012.0016), *Nasutoceratops* (UMNH VP 16800), *Pentaceratops* (OMNH 10165), and *Triceratops* (USNM 2416, 5420), but distinct from *Diabloceratops* (UMNH VP 16699), which possess solid postorbital horncore bases (Kirkland & DeBlieux, 2010). This condition in *Albertaceratops* is unknown. The placement of the transverse buttress caudal to the postorbital horncore, and incorporation of only the rostral chamber into the horncore are distinct from the condition in all other ceratopsids (e.g., *Coronosaurus*: TMP 2002.068.0012, *Pachyrhinosaurus*: TMP 1989.055.0021; *Triceratops*: USNM 2416). This latter condition is similar to that in centrosaurines (e.g., *Pachyrhinosaurus lakustai, Einiosaurus, Achelousaurus*), in which both rostral and caudal chamber may enter the horncore bases, and distinct from chasmosaurines (e.g., *Anchiceratops, Eotriceratops, Pentaceratops, Triceratops*), in which only the caudal chamber enters the horncore (Farke, 2010). Although highly variable (Farke, 2010), the rostral chamber in centrosaurine taxa tends to be equal to (*Centrosaurus*—TMP 1986.018.0087/0101, 2002.068.0012; *Coronosaurus* TMP 2002.068.0018/0030) or larger than (*Centrosaurus*—TMP 1965.012.0005, TMP 1980.018.0083; *Coronosaurus*—TMP 2002.068.0005; *Pachyrhinosaurus lakustai*—TMP 1989.055.0808/1131; *Styracosaurus*—1998.093.0064) the caudal chamber, whereas in chasmosaurines the caudal chamber is generally the larger of the two (*Anchiceratops*—TMP 1984.012.0016; *Triceratops* USNM 5740).

The partial transverse section along the frontal suggest a vaulted skull roof, similar to that of basal centrosaurines (e.g., *Albertaceratops*, *Diabloceratops, Nasutoceratops*) and some chasmosaurines (Ryan, 2007; Kirkland & DeBlieux, 2010; Sampson et al., 2010; Lund, Sampson & Loewen, 2016).

#### TMP 2017.022.0037

Given the small size and unfused nature of TMP 2017.022.0037 it likely represented a subadult individual, and is less distinct in its morphology than TMP 1989.068.0001. Due to its incompleteness, the specimen preserves no evidence of a roofed supracranial sinus (as in TMP 1989.068.0001), nor other diagnostic synapomorphies for Ceratopsidae. As a result, although considered here to pertain to Ceratopsidae, the specimen cannot by confidently diagnosed beyond Ceratopsoidea. The specimen is most similar to that of an indeterminate subadult horncore previously referred to *Albertaceratops* TMP 2002.069.0001 (Ryan, 2007; Ryan, Russell & Hartman, 2010), but represents a slightly larger, possibly older, animal. Features such as the dorsal position over the orbit, and the straight, dorsolaterally projecting aspect of the horncore are shared with TMP 1989.068.0001, and suggest that it may represent an ontogenetically younger individual of the same taxon. Lack of developed cornual diverticulum of the supracranial sinus entering the postorbital is not a problem for this assessment, as this has been shown to develop ontogenetically in many ceratopsid taxa (Farke, 2010).

## Discussion

*Referral:* Amongst centrosaurines, *Albertaceratops*, *Diabloceratops*, *Nasutoceratops*, and *Machairoceratops* possess long postorbital horncores (Ryan, 2007; Kirkland & DeBlieux, 2010; Sampson et al., 2013; Lund et al., 2016; Lund, Sampson & Loewen, 2016), and a long-horned postorbital inferred as plesiomorphic for Centrosaurinae, and inferred presence in *Xenoceratops* and *Wendiceratops* (Ryan, Evans & Shepherd, 2012; Evans & Ryan, 2015). Due to a poorer fossil record, our understanding of the plesiomorphic condition in chasmosaurines is less clear, with many early occurring (and basally branching taxa) showing modest horns (Sampson et al., 2010), and other taxa represented by fragmentary material (Longrich, 2013). The only known ceratopsid species from the Foremost Formation is *Xenoceratops foremostensis* (Ryan, Evans & Shepherd, 2012), with other taxa (with the potential exception of *Diabloceratops* and *Machairoceratops*) occurring in formations from later time intervals (Ryan, 2007; Sampson et al., 2013; Evans & Ryan, 2015; Lund et al., 2016).

Although not currently diagnostic to subfamily, several features suggest that TMP 1989.068.0001 may be derived from a centrosaurine, and not a chasmosaurine. These include the rostral chamber of the supracranial sinus being larger than the caudal chamber, and the rostral chamber forming the cornual diverticulum. Given the evidence for non-overlapping temporal and geographic ranges in centrosaurine taxa throughout the later units of the Belly River Group (Ryan & Russell, 2005; Ryan, Holmes & Russell, 2007; Ryan et al., 2012; Brown, 2013; Mallon et al., 2013; Evans & Ryan, 2015), and similar patterns across western North America as a whole (Horner, Varricchio & Goodwin, 1992; Sampson, 1995; Lund et al., 2016), the co-occurrence of the postorbitals with *Xenoceratops foremostensis* must be considered.

The hypodigm and referred specimens of *Xenoceratops* are limited to elements of the parietosquamosal frill, and a nasal fragment, but Ryan, Evans & Shepherd (2012) also reference a fragmentary skull from the Foremost Formation that may be referable to this taxon. Although not described in detail or figured, this specimen preserves “portions of two large-diameter, elongate postorbital horncores” (Ryan, Evans & Shepherd, 2012, p. 1260). This morphology is broadly consistent with that preserved in TMP 2017.022.0037 and 1989.068.0001, as well as other basal centrosaurines (e.g., *Albertaceratops*). Although the described isolated postorbitals may be assignable to *Xenoceratops*, they are here regarded as Ceratopsidae indeterminate.

*Implications:* The two postorbitals described here represent some of the earliest ceratopsid remains, and indicate a form with clearly massive, dorsolaterally projecting, straight postorbital horncores. If assignable to *Xenoceratops*, or other centrosaurine taxon, they represent another occurrence of long-horned morphology in basal Centrosaurinae, further supporting evidence of this as the plesiomorphic condition for this subfamily. Although our understanding of middle Campanian chasmosaurines is limited, a pattern continues to emerge in which the postorbital horns of many centrosaurines (e.g., *Albertaceratops, Nasutoceratops*) greatly exceed those of their contemporary chasmosaurine counterparts (e.g., *Judiceratops*), a situation that is reversed by the late Campanian. This reversal is accomplished due to both an evolutionary reduction in the postorbital horncore within the Centrosaurinae lineage, and an exaggeration within the Chasmosaurinae lineage. Despite this reversal, it is not until taxa such as *Pentaceratops* (late Campanian) and *Anchiceratops* and *Arrhinoceratops* (latest Campanian/early Maastrichtian) that Chasmosaurinae evolve postorbital horns larger than those of some basal Centrosaurinae.

Although the amount of material represented by the present description is modest, it does add to our growing understanding of early evolution and diversification of Ceratopsidae. Specifically, TMP 1989.068.0001 adds to our understanding of the morphology of the supracranial sinus and cornual diverticula in basal Ceratopsidae, and their evolution within Chasmosaurinae and Centrosaurinae. This structure in basal Centrosaurinae has been problematic, as it is both difficult to assess in taxa with articulated/complete skulls (e.g., *Diabloceratops*, *Albertaceratops*, *Nasutoceratops*), and not preserved in other taxa (e.g., *Xenoceratops*, *Wendiceratops*). The results here suggest the formation of the cornual diverticulum in this long-horned morph is distinct from those seen in better documented late Campanian/Maastrichtian Chasmosaurinae (Farke, 2010).

The last decade has seen a tremendous increase in our understanding of the evolution and diversification of Ceratopsidae, at least in part due to the discovery of many new basal, and earlier occurring, taxa (Ryan, 2007; Kirkland & DeBlieux, 2010; Ryan, Evans & Shepherd, 2012; Sampson et al., 2013; Evans & Ryan, 2015; Lund, Sampson & Loewen, 2016), and potential sister taxa (Wolfe & Kirkland, 1998; Farke et al., 2009; Sues & Averianov, 2009a; Sues & Averianov, 2009b; Wolfe et al., 2010) to this clade. Despite these advances, a significant temporal and morphological gap still exists between the Turonian-aged *Zuniceratops* and Campanian-aged early ceratopsids such as *Albertaceratops*, *Diabloceratops, Xenoceratops* and *Judiceratops* (Ryan, 2007; Kirkland & DeBlieux, 2010; Wolfe et al., 2010; Ryan, Evans & Shepherd, 2012; Longrich, 2013; Campbell, 2014). Continued work finding and documenting new discoveries will undoubtedly fill in this gap and likely offer further surprises in the evolutionary history of this last great radiation of dinosaurs.

## Anatomical Abbreviations

ali: alisphenoid fossa
cc: caudal chamber of supracranial sinus
ff: frontal fontanelle
lg: longitudinal grooves
pal: palpebral
po: postorbital
poh: postorbital horncore
orb: orbit
rc: rostral chamber of supracranial sinus
ssf: sutural surface for frontal
sslac: sutural surface for lacrimal
ssls: sutural surface for laterosphenoid
sspal: sutural surface for palpebral
sssq: sutural surface for squamosal
tb: transverse buttress of supracranial sinus

## Orientation Abbreviations

c: caudal
d: dorsal
n: north
m: medial

## Institutional Abbreviations

CMN: Canadian Museum of Nature, Ottawa
MOR: Museum of the Rockies, Bozeman
MSM: Mesa Southwest Museum, Mesa
OMNH: Oklahoma Museum of Natural History, Norman
TMP: Royal Tyrrell Museum of Palaeontology, Drumheller
ROM: Royal Ontario Museum, Toronto
UMNH: Natural History Museum of Utah, Salt Lake City
USNM: National Museum of Natural History, Smithsonian, Washington
ZIN PH: Paleoherpetological Collection, Zoological Institute, Russian Academy of Sciences, Saint Petersburg

## Additional Information and Declarations

### Field Study Permissions

The following information was supplied relating to field study approvals (i.e., approving body and any reference numbers): A permit for excavation and collection of vertebrate fossil resources was approved by Alberta Culture and Tourism and the Royal Tyrrell Museum of Palaeontology—(Permit to Excavate Palaeontological Resources 17-017).

### Data Availability

The following information was supplied regarding data availability: The specimens described are housed at the Royal Tyrrell Museum of Palaeontology.
